# Precipitation Mediates the Response of Carbon Cycle to Rising Temperature in the Mid-to-High Latitudes of the Northern Hemisphere

**DOI:** 10.1371/journal.pone.0132663

**Published:** 2015-07-14

**Authors:** Xin Lin, Junsheng Li, Jianwu Luo, Xiaopu Wu, Yu Tian, Wei Wang

**Affiliations:** 1 College of Water Sciences, Beijing Normal University, Beijing, China; 2 State Key Laboratory of Environmental Criteria and Risk Assessment, Chinese Research Academy of Environmental Sciences, Beijing, China; Fudan University, CHINA

## Abstract

Over the past decades, rising air temperature has been accompanied by changes in precipitation. Despite relatively robust literature on the temperature sensitivity of carbon cycle at continental to global scales, less is known about the way this sensitivity is affected by precipitation. In this study we investigate how precipitation mediates the response of the carbon cycle to warming over the mid-to-high latitudes in the Northern Hemisphere (north of 30°N). Based on atmospheric CO_2_ observations at Point Barrow (BRW) in Alaska, satellite-derived NDVI (a proxy of vegetation productivity), and temperature and precipitation data, we analyzed the responses of carbon cycle to temperature change in wet and dry years (with precipitation above or below the multiyear average). The results suggest that, over the past three decades, the net seasonal atmospheric CO_2_ changes at BRW were significantly correlated with temperature in spring and autumn, yet only weakly correlated with temperature and precipitation during the growing season. We further found that responses of the net CO_2_ changes to warming in spring and autumn vary with precipitation levels, with the absolute temperature sensitivity in wet years roughly twice that in dry years. The analyses of NDVI and climate data also identify higher sensitivity of vegetation growth to warming in wet years for the growing season, spring and summer. The different temperature sensitivities in wet versus dry years probably result from differences in soil moisture and/or nutrient availability, which may enhance (inhibit) the responsiveness of carbon assimilation and/or decomposition to warming under high (low) precipitation levels. The precipitation-mediated response of the terrestrial carbon cycle to warming reported here emphasizes the important role of precipitation in assessing the temporal variations of carbon budgets in the past as well as in the future. More efforts are required to reduce uncertainty in future precipitation projections, and to better represent the nonlinearity of carbon cycle responses to climate in current state-of-the-art land surface models.

## Introduction

The terrestrial biosphere is a key component controlling numerous processes and feedback loops within the climate system. It is not only of major relevance for carbon cycles, but also impacts water and energy exchanges between land and atmosphere. Terrestrial ecosystems currently remove roughly 1/4 of global annual anthropogenic CO_2_ emissions [1]. Predicting future land carbon sink is challenging, partly due to our limited understanding of the response of the terrestrial carbon cycle to climate change. The IPCC climate-carbon coupling models predict that future climate change will decrease global land carbon sink, but large uncertainties exist in the magnitude [2]. Reducing this uncertainty is essential to more accurate future climate predictions.

The mid-to-high latitude continental regions of the Northern Hemisphere (abbreviated as NH hereafter) are currently considered to be a main contributor of global land carbon sink, as indicated by atmospheric inverse modeling and forest inventory data [3–6]. Continued satellite observations during the past 30 years also show a significant increase in NDVI over this region [7–9]. Several studies have linked this enhanced vegetation growth and carbon sink with the rapid warming over the mid-to-high latitude regions [10,11]. For example, using a process based ecosystem model, Lucht *et al* [10] suggested that temperature change fully explained the vegetation greening trend in the NH. A significant and positive correlation between temperature and NDVI was also observed over the NH at the continental scale according to several satellite-based studies [9,12–14]. Keeling *et al* [15] also found that the amplitude of CO_2_ concentration at Point Barrow, Alaska significantly increased in response to the rising temperature, although the effect of temperature change on the carbon cycle of the NH varied in different seasons [16,17].

Despite the relatively robust literature on the response of carbon cycle to changing temperature over the NH at the continental scale, some questions remain unsolved. How do precipitation changes affect the temperature sensitivity of the carbon cycle and its components in the NH? Does the effect of precipitation changes on the temperature sensitivity of the carbon cycle vary across different seasons? In the past several decades, rising temperature has been accompanied by changes in precipitation, and this trend will continue in the future [18]. Hence, a better understanding of the questions above is critical to predict the evolution of the future carbon cycle over the NH. In this study, we used 30-year time series datasets of atmospheric CO_2_ observations at Point Barrow (BRW), Alaska and satellite-derived NDVI along with corresponding temperature and precipitation data to investigate the effect of precipitation changes on the temperature sensitivity of the carbon cycle in the NH.

## Data and Methods

### Data

Long-term time series of atmospheric CO_2_ concentrations have been widely used to investigate changes in the carbon cycle and the underlying mechanisms (e.g., refs [15,17,19–22]). Point Barrow station (BRW– 71.32°N, 156.61°W, 11.00 m.a.s.l.) in Alaska, with its atmospheric CO_2_ records since 1979, is recognized as a global background atmospheric station that samples air masses representative of the mid-to-high latitudes of the NH [23,24]. Previous studies showed that the CO_2_ seasonality at BRW and its changes over the past decades were mainly influenced by the ecosystem fluxes in Arctic and boreal regions, as well as in temperate regions [20,25]. Therefore we confined our analyses to the mid-to-high latitudes of the NH north of 30°N in this study.

The monthly averaged atmospheric CO_2_ concentrations at BRW, based on continuous *in-situ* CO_2_ measurements, were obtained from the Earth System Research Laboratory of the National Oceanic and Atmospheric Administration (NOAA/ESRL) network for the period of 1979–2009 (http://www.esrl.noaa.gov/gmd/ccgg/globalview). The CO_2_ time series is a combination of three signals: an interannual trend, a seasonal cycle, and a residual [25,26]. Following the approach described in ref.[26], the interannual trend, which is mainly driven by anthropogenic forcing (e.g., fossil fuel combustion, deforestation), was removed by applying curve-fitting procedures (the CCGVU program) with a polynomial curve of degree 2, four harmonic function, and time-filtered residuals. The detrended CO_2_ time series (the harmonics plus the residuals) were extracted from the monthly atmospheric CO_2_ concentrations and used in subsequent analyses.

Normalized difference vegetation index (NDVI), defined as the ratio of the difference between near-infrared reflectance and red visible reflectance to their sum, is a remote-sensing-derived vegetation index to measure vegetation greenness and photosynthetic activity [12,27]. The time series of NDVI data used in this study were derived from the AVHRR/NOAA satellite and produced by the Global Inventory Monitoring and Modeling Studies (GIMMS) group, at a spatial resolution of 0.083° and 15-day interval for the period of 1982–2009 [27]. The GIMMIS NDVI datasets have been calibrated to minimize the effects of orbital drift, cloud cover, solar angle, volcanic eruption and other atmospheric contaminations [9,28]. To reduce the noise in NDVI data, we produced monthly NDVI data from two images of each month by applying the Maximum Value Composite (MVC) method [29].

The monthly temperature and precipitation data covering the study period were obtained from the Climatic Research Unit (CRU) TS 3.1 datasets at the resolution of 0.5° [30].

### Analyses

To investigate how interannual variations of precipitation mediate the responsiveness of carbon cycle to temperature changes over the NH, we estimated and differentiated the temperature sensitivity of the net CO_2_ uptake/release in the NH (indicated by the net changes in detrended CO_2_ time series at BRW) over the entire observing period of 1979–2009 and during wet and dry years respectively. The analyses were performed for the growing season, as well as for spring, summer, and autumn to examine whether the effect of precipitation changes on the temperature sensitivity of vegetation growth varies across different seasons. For each year, we calculated the changes in detrended atmospheric CO_2_ concentrations between the start and the end of the growing season, spring, summer, and autumn, as indicative of the net CO_2_ uptake/release during the corresponding period. Since the duration of the atmospheric CO_2_ time series is limited to 31 years, wet (dry) years are simply defined as years of positive (negative) anomalies based on the total precipitation amount during the growing season or each season. For the specific focused season and years, the temperature sensitivity was calculated by fitting a least-square linear regression model (Eq 1):

y=a+bx+ε (1)

where y represents the net CO_2_ change at BRW for the growing season or each season, x is the corresponding temperature, a and b are the regression coefficients (a: intercept; b: the temperature sensitivity of the net CO_2_ change for the specific season and years), and ε is the residual random error. A least-square linear regression model is used to derive the regression coefficients (i.e. a and b). A p-value less than 0.05 is considered significant. To further investigate how precipitation mediates the response of vegetation productivity to temperature changes, we estimated the temperature sensitivity of satellite-derived NDVI in the same way for the entire observing period of 1982–2009 and for wet and dry years, respectively.

In this study, the growing season is defined as the period from April to October, spring as from April to May, summer as from June to August, and autumn as from September to October [9,31,32]. It should be noted that the actual growing season may differ from the definition and vary across the whole study area. Based on the definitions, we calculated the average NDVI and climate variables for the growing season and each of the three seasons respectively. Pixels with the multi-year average growing season NDVI less than 0.1 are considered as non-vegetated areas and excluded from this study [32].

## Results

### Temperature sensitivity of carbon balance vs. precipitation

**Fig 1:**
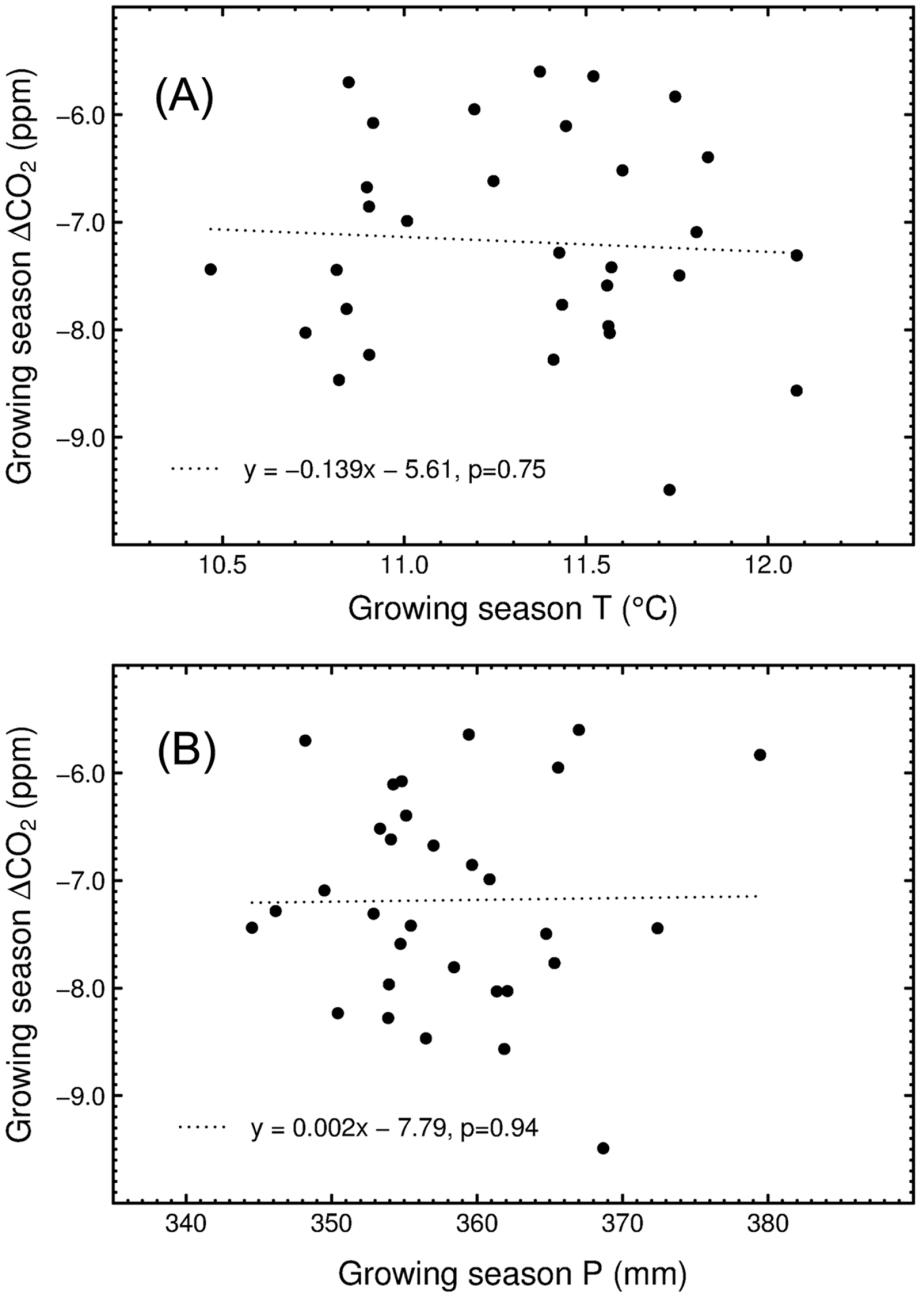
Responses of the net changes in the growing season atmospheric CO_2_ concentrations (abbreviated as ΔCO_2_) at Point Barrow, Alaska to climate in 1979–2009. (A) The relationships between ΔCO_2_ and the growing season temperature. (B) The relationships between ΔCO_2_ and the growing season precipitation. The dotted lines were produced from linear regressions of ΔCO_2_ versus the growing season temperature and precipitation, which are not significant at a level of p<0.05 (p = 0.75 and p = 0.94, respectively). The growing season temperature and precipitation were averaged over the NH north of 30°N.

Firstly, to investigate how the carbon balance in the NH terrestrial ecosystems was regulated by individual climatic variables over the past three decades, we analyzed the net change in the atmospheric CO_2_ concentrations at BRW between the onset and the end of the growing season for each year during 1979–2009 and its relationship with temperature and precipitation respectively. As illustrated in Fig 1, the northern terrestrial ecosystems north of 30°N behave as a net carbon sink during the growing season, but the magnitude of the net growing season CO_2_ uptake is not significantly correlated with the growing season temperature or precipitation (p = 0.75 and p = 0.94, respectively).

**Fig 2:**
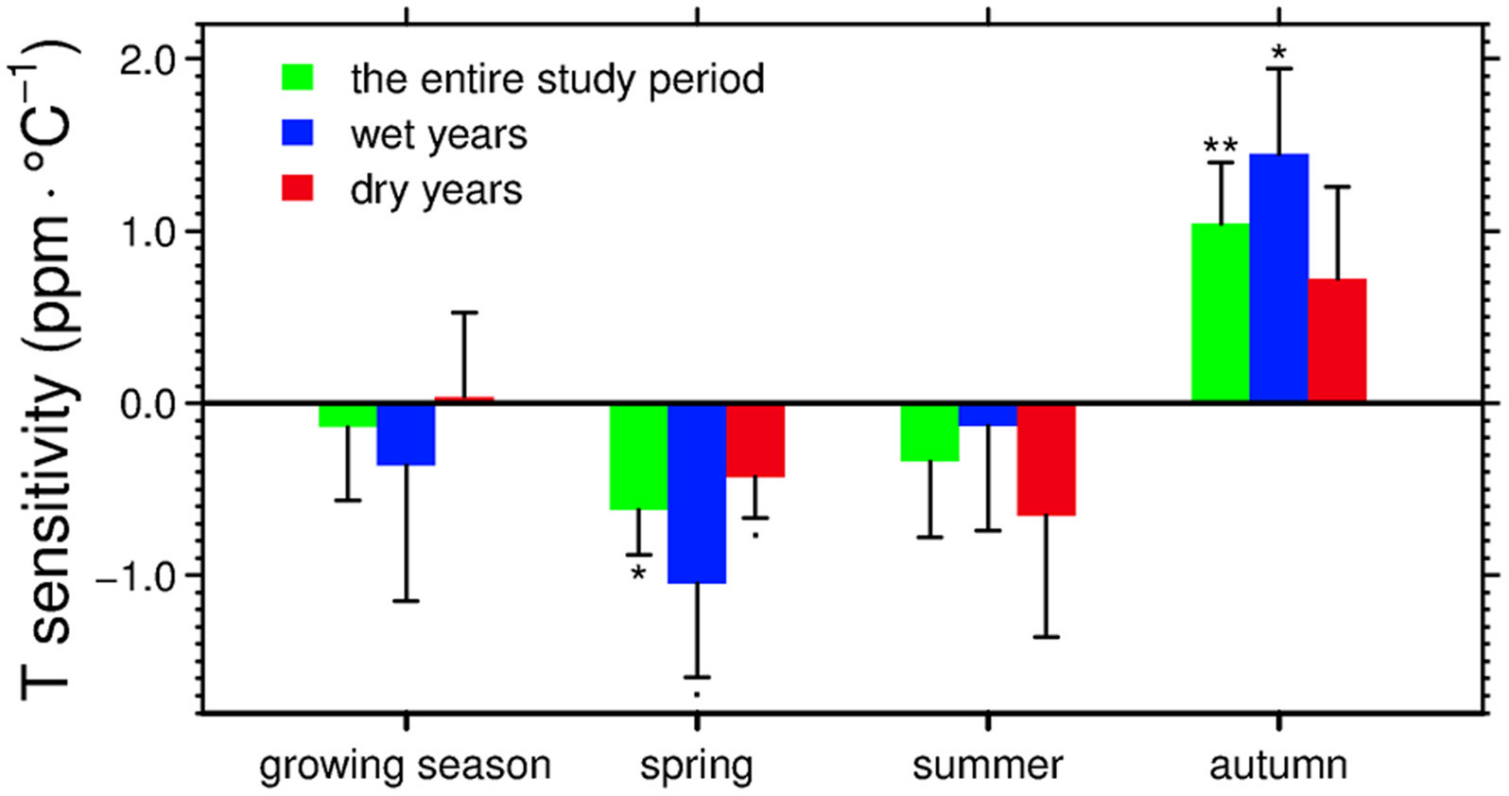
Temperature sensitivity of the net seasonal changes in atmospheric CO_2_ concentrations at Point Barrow, Alaska (abbreviated as ΔCO_2_) over the period of 1979–2009. The error bar represents one standard error of the temperature sensitivity produced from a linear regression of ΔCO_2_ versus temperature. Annotations of asterisks or dots indicate the significance levels of linear regressions (“**” for p<0.01, “*” for p<0.05 and “.” for p<0.1). Wet and dry years are simply defined as years of positive and negative precipitation anomalies, as described in *Data and Methods*. The growing season and seasonal temperature and precipitation were averaged over the NH north of 30°N.

To better understand the underlying ecosystem processes that drive the carbon cycle in spring, summer and autumn, we analyzed seasonal changes in the atmospheric CO_2_ concentrations and their linkages with seasonal temperature and precipitation. In both spring and summer, the northern terrestrial ecosystems sequester atmospheric CO_2_. The net changes in atmospheric CO_2_ concentrations are negative in 24 and 31 out of the entire 31 years for spring and summer respectively. Over the period of 1979–2009, the strength of the net spring CO_2_ uptake significantly increased in warmer conditions (p = 0.02), with the sensitivity of -0.62 ppm·°C^-1^ (Fig 2). The magnitude of the net summer CO_2_ uptake also increased with temperature but not significantly (p = 0.45; Fig 2). When further examining how the spring carbon balance responds to temperature changes for wet and dry years respectively, we find that much higher temperature sensitivity of net spring CO_2_ uptake (in absolute values) is observed during wet years (-1.05 ppm·°C^-1^, p = 0.08) than dry years (-0.43 ppm·°C^-1^, p = 0.09; Fig 2), although the correlations between the net spring CO_2_ uptake and temperature are marginally significant for both. In contrast with spring and summer during which photosynthetic CO_2_ uptake exceeds heterotrophic respiration, in autumn, the northern terrestrial ecosystems become a net carbon source to atmosphere. Over the entire 31 years, the magnitude of the net release of CO_2_ in autumn significantly increased with temperature, with the sensitivity of 1.041 ppm·°C^-1^ (p = 0.01). This phenomenon is consistently found when analyzing years of high versus low precipitation levels, with the temperature sensitivity during wet years (1.44 ppm·°C^-1^, p = 0.01) twice as high as that during dry years (0.72 ppm·°C^-1^, p = 0.20; Fig 2).

### Temperature sensitivity of vegetation growth vs. precipitation

**Fig 3:**
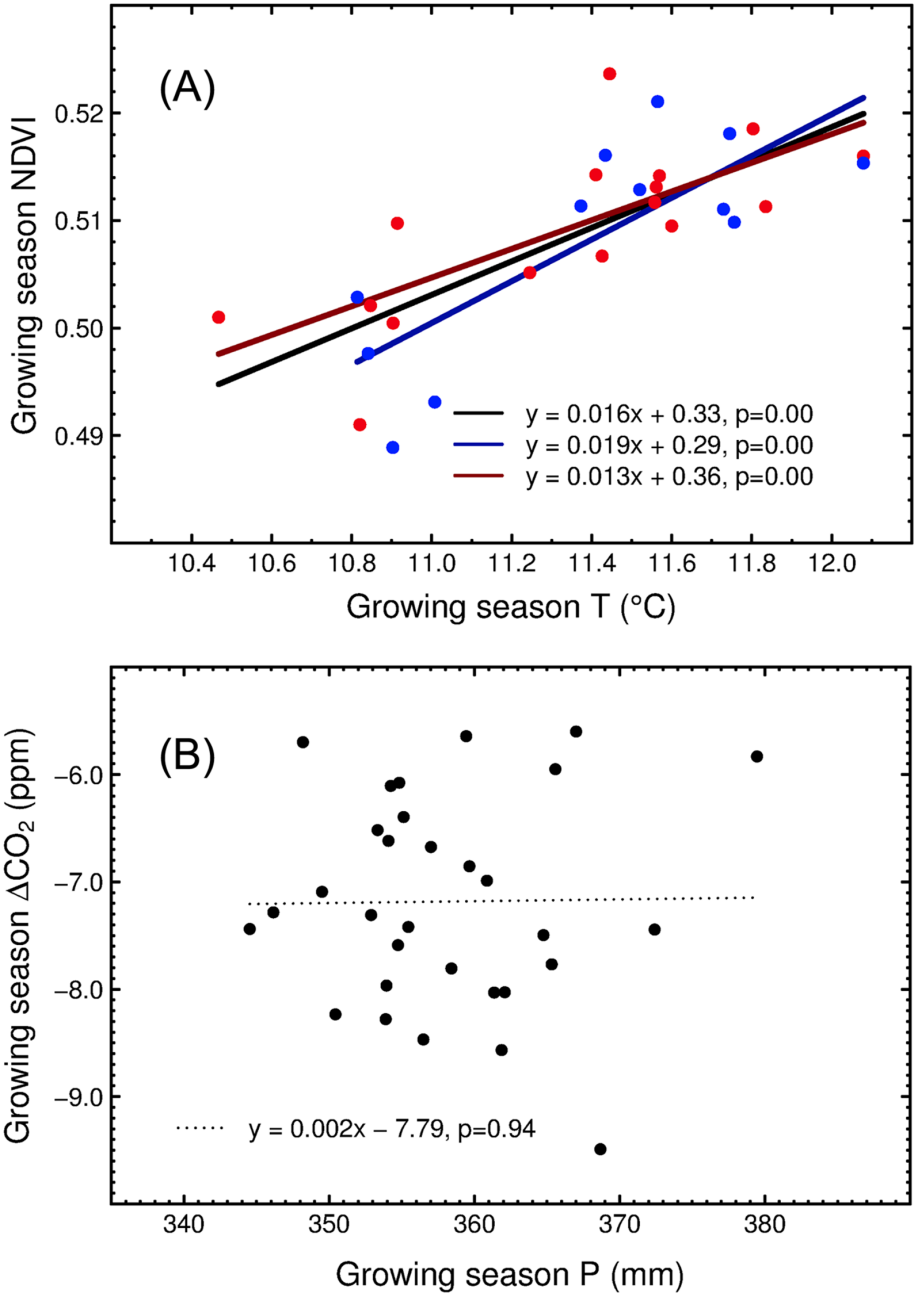
Responses of the growing season NDVI to climate over the Northern Hemisphere north of 30°N in 1982–2009. (A) The relationships between the growing season NDVI and the growing season temperature. (B) The relationships between the growing season NDVI and the growing season precipitation. Blue and red points in (A) represent observations during wet and dry years respectively. The solid lines were produced from linear regressions of the growing season NDVI versus the growing season temperature for the entire 28 years (black), wet years (blue) and dry years (red) respectively, all of which are significant (p<0.01). The dotted line in (B) was produced from a linear regression of the growing season NDVI versus the growing season precipitation for the period 1982–2009, which is not significant at a level of p<0.05 (p = 0.49). Wet and dry years are simply defined as years of positive and negative precipitation anomalies, as described in *Data and Methods*.

As vegetation productivity dominates the carbon flux between the northern biosphere and atmosphere, we applied similar analyses to the growing season NDVI (a proxy of vegetation productivity) to investigate the different responses of vegetation growth to temperature changes during wet years and dry years. Fig 3 illustrates the relationship between the growing season NDVI and the growing season temperature or precipitation between 1982 and 2009 over the mid-to-high NH north of 30N°. As shown in Fig 3, the growing season NDVI increased in response to rising temperature with the sensitivity of 0.016±0.003°C^-1^(R = 0.74, p<0.01; Fig 3A), while it was not significantly correlated with precipitation (R = 0.14, p = 0.49; Fig 3B). Although no direct linkage is found between the growing season NDVI and precipitation, when we divide the entire observing period into wet years and dry years, there is a notable difference between the two periods in the temperature sensitivity of vegetation growth. During wet years, vegetation growth is more sensitive to temperature changes compared to dry years, with the temperature sensitivity of 0.019±0.005°C^-1^ (R = 0.79, p<0.01) and 0.013±0.003°C^-1^ (R = 0.73, p<0.01) respectively (Fig 3A).

**Fig 4:**
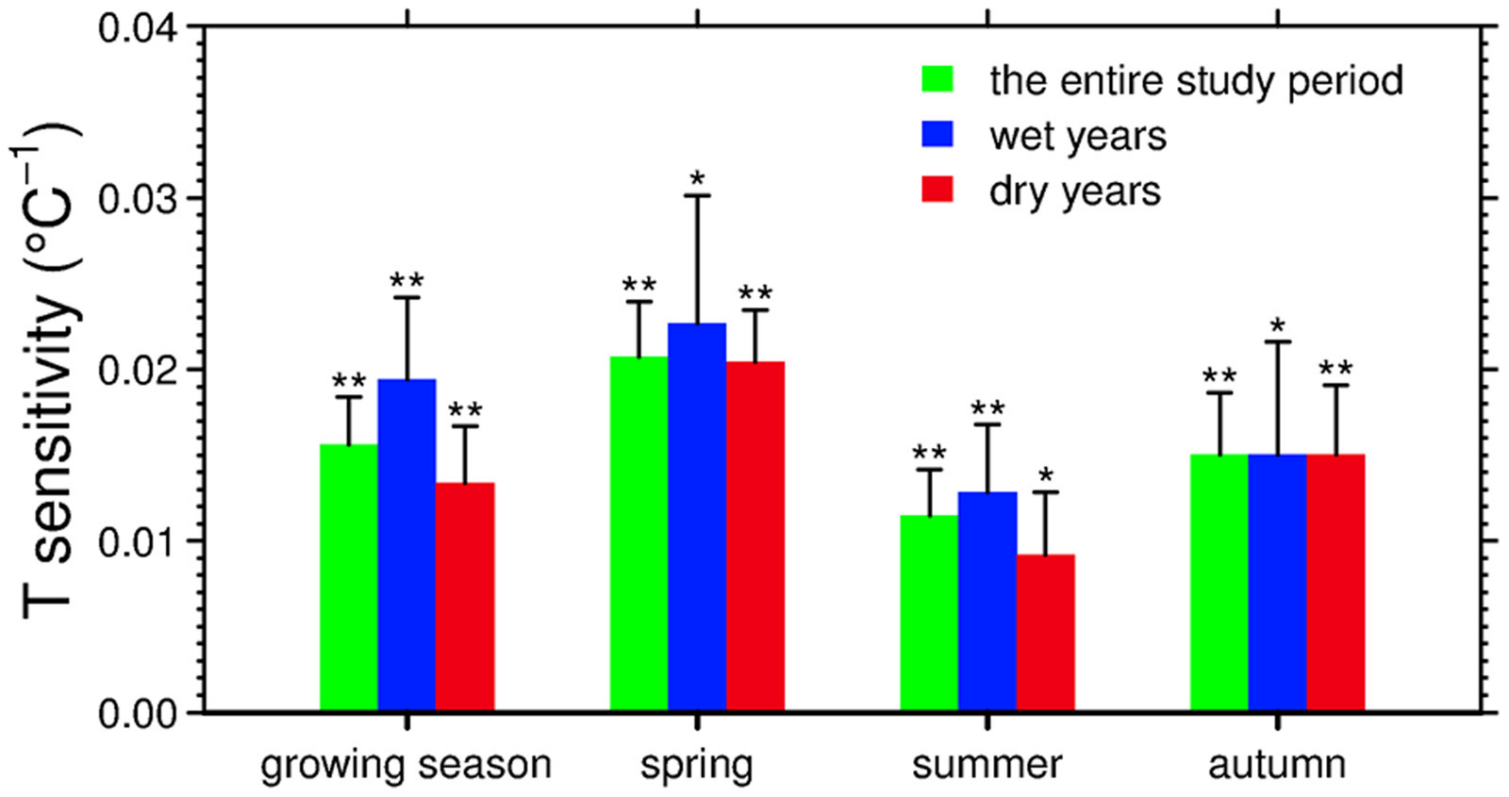
The temperature sensitivity of NDVI over the Northern Hemisphere north of 30°N over the period of 1982–2009. The error bar represents one standard error of the temperature sensitivity produced from a linear regression of NDVI versus temperature. Annotations of asterisks indicate the significance levels of linear regressions (“**” for p<0.01 and “*” for p<0.05). Wet and dry years are simply defined as years of positive and negative precipitation anomalies, as described in *Data and Methods*.

Precipitation-mediated effects on temperature sensitivity are also found when we examine how seasonal vegetation growth responds to climate factors. As shown in Fig 4, during the entire study period, the seasonal NDVI increased in warmer conditions across all three seasons, with the sensitivity of 0.021±0.003°C^-1^, 0.011±0.003°C^-1^, and 0.015±0.004°C^-1^ for spring, summer, and autumn, respectively. Although the positive seasonal NDVI-temperature relationships are consistently detected, there are notable contrasts between wet years and dry years in the temperature sensitivity of vegetation growth. In both spring and summer, vegetation growth is more sensitive to temperature during wet years than during dry years. The sensitivity of spring vegetation growth to temperature is 0.023±0.007°C^-1^ for wet years in comparison with 0.020±0.003°C^-1^ during dry years (Fig 4). In summer, despite a smaller magnitude of temperature sensitivity, a considerable difference is also documented between wet years (0.013±0.004°C^-1^) and dry years (0.009±0.004°C^-1^) (Fig 4). By contrast, in autumn, the temperature sensitivities of vegetation growth are almost the same (0.015±0.007°C^-1^ and 0.015±0.004°C^-1^) for wet and dry years (Fig 4).

## Discussion

The responses of carbon balance to temperature changes are dependent on the balance between vegetation productivity and ecosystem respiration, both of which are temperature sensitive [33–35]. In this study, we find that there is no statistically significant correlation between the growing season temperature and the change in atmospheric CO_2_ concentrations at BRW during the past three decades (Fig 1A), although the growing season NDVI is significantly and positively correlated with the growing season temperature over the NH (Fig 3A). This suggests that rising temperature enhances vegetation growth over the NH but does not necessarily increase the net carbon uptake of terrestrial ecosystems, since warming will accelerate decay of soil organic matter, thereby leading to the release of CO_2_ to the atmosphere [36]. This result does not support the results of previous climate-carbon coupling model analyses by Qian *et al* [37], who suggested that future intense warming in the NH will enhance the land carbon sink. In contrast, 3 of 10 carbon cycle models used for the IPCC 5^th^ Assessment Report showed a significant but negative correlation between carbon sink and temperature over the NH, while the other models indicated that the interannual variations of carbon sink are not significantly related to temperature [38].

There are now substantial observational and modeling studies demonstrating that the effects of temperature changes on the carbon cycle vary across different seasons [16,17,39,40]. For example, it has been suggested that a warmer spring and the associated earlier vegetation green-up date generally tend to stimulate vegetation productivity more than ecosystem respiration, leading to an increase in the net carbon uptake in the NH [15]. In contrast, rising autumn temperature decreases net carbon uptake [17,39], although the mechanisms are still poorly understood [40]. The opposite responses of carbon balance to spring and autumn temperature are also supported by our study. We find that over the period of 1979–2009, 1°C of rising autumn temperature over the NH tended to increase the autumn atmospheric CO_2_ concentration at BRW by 1.041 ppm·°C^-1^, which was about 70% higher than the sensitivity in spring. Furthermore, our results also suggest that, although the autumn temperature sensitivity of vegetation growth during wet years is similar to that during dry years, the temperature sensitivity of the change in the autumn atmospheric CO_2_ concentration at BRW in dry years is only 50% that of wet years. This result not only implies that the temperature sensitivity of autumn ecosystem respiration is strongly regulated by precipitation conditions, but also suggests that the temperature sensitivity of autumn ecosystem respiration in wet years is higher than in dry years [41]. Several field experiments have pointed out that soil respiration becomes more sensitive to temperature in response to rising soil moisture contents [42,43].

The differential temperature sensitivity mediated by precipitation is also found for vegetation growth in the NH north of 30°N during the past three decades. Our analyses confirms that temperature plays a dominant role in driving the overall trends in vegetation greenness over the mid-to-high latitudes of the NH [9,10,12–14,44], while the contribution of precipitation is limited [14,45,46]. However, the sensitivity of vegetation growth to temperature changes is not constant but dependent on precipitation levels: during wet years with precipitation above the average, the warmer climate stimulates a higher rate of vegetation growth, probably as a result of abundant soil moisture and nutrient availability that may accelerate photosynthesis in concert with rising temperature [47]; during dry years with precipitation below the average, the enhancement of vegetation growth in response to warming is compromised by limited water supply, probably through soil moisture deficits and locally intensified drought stress that may lead to decreased plant photosynthesis and changes in carbon allocation [48,49]. Unlike temperature that exerts overall impacts on northern vegetation growth directly through enhancing photosynthesis and extending the growing season, precipitation influences vegetation growth indirectly by mediating its responsiveness to temperature changes. This precipitation-mediated effect on the temperature sensitivity suggests a non-linear response of northern vegetation growth to climate change during the past three decades.

The non-linear responses of vegetation growth to temperature mediated by precipitation is not only observed for the entire growing season, but also consistently found for spring and summer. There is a substantial decrease in the temperature sensitivity of seasonal vegetation growth during dry years compared to wet years, notably, by as much as *c*.*a*. 30% for summer. The weakened responsiveness of vegetation growth to temperature may be attributed to a regional decline in vegetation growth under seasonal drought stress, which is a result of lower local precipitation as well as warming-induced evaporative drying [50]. In summer when temperature is high, the low soil moisture would be particularly stressful to plant growth without sufficient water supply from precipitation [51]. Previous satellite-derived analyses at northern high latitudes confirmed the vegetation browning in response to summer drought at both continental and regional scales [19,50,52,53]. In addition, evidence in support of the reduction in plant growth associated with summer drought also comes from satellite-driven models [54], CO_2_ flux measurements [55], forest biomass inventory [56], and tree ring width measurements [48,51]. In autumn, on the contrary, the temperature sensitivity of vegetation growth does not show too much difference between wet and dry years, probably implying that factors other than temperature and precipitation may limit vegetation growth in autumn, such as solar radiation, day length, and photoperiod [40,57,58].

A few caveats should be noted when interpreting the results. First, our analyses show that rising temperature is the primary factor driving the overall trend of vegetation growth in the mid-to-high latitudes of the NH. However, other factors may be more important when we investigate specific vegetation types and climate regimes. For instance, Zhao and Running (2010) [59] reported that for northern mid-latitudes (22.5–47.5°N), the growing season precipitation rather than temperature controlled net primary productivity over the period of 2000–2009. The authors also noted that this region is covered with large areas of temperate grasslands and croplands, where plant growth is limited by water availability [47,60,61]. This implies that the dominant factor driving variations in vegetation growth may depend on the scale we are focusing on and vary across different ecosystems and climate regimes.

Second, although the differential temperature sensitivity of plant growth and carbon balance mediated by precipitation may be linked to differences in soil moisture and/or nutrient availability as supported by several field studies (e.g., [62–66]), direct verification of this linkage at large scales is difficult due to the lack of long-term, spatially resolved, and biophysically relevant soil datasets. For example, the soil moisture measurements from satellite microwave sensors retrieve only the soil moisture up to the top 1–1.5cm of the soil and are most accurate in areas of low vegetation density [67], whereas the profile soil moisture datasets produced from the Gravity Recovery and Climate Experiment (GRACE) satellite products cover a relatively short time period since the middle of 2002 (e.g., [68]). Datasets of nutrient availability (e.g., soil N/P content) are also not readily available with sufficient spatial resolution and temporal coverage, impeding our understanding of mechanisms underlying the precipitation-mediated effects on responsiveness of the carbon cycle to rising temperature. This highlights the urgent need to develop long-term and high-quality soil datasets, which rely on comprehensive soil monitoring networks, both on the ground and from satellites. Besides, field studies in different climate regimes and ecosystems are required to investigate how changes in soil moisture and/or nutrients regulate the vital processes of plant growth and carbon cycle.

## Conclusions

Part of the uncertainty in predicting land carbon sinks arises from our limited understanding of the complex feedbacks between the terrestrial carbon cycle and climate systems. Based on time-series datasets of the atmospheric CO_2_ concentration at BRW, satellite-derived NDVI, and the corresponding temperature and precipitation during the past three decades, our analyses show that changes in carbon balance and vegetation growth over the mid-to-high latitudes of the NH in response to the recent warming is dependent on precipitation levels. The precipitation-mediated nonlinear responses of the terrestrial biosphere to warming, on the one hand, emphasize the important role precipitation plays in evaluating the temporal variations of carbon budgets, presenting a need to reduce uncertainties in precipitation projections given the substantial uncertainty among climate models in future precipitation projections [18]. On the other hand, field and modeling experiments that examine how ecosystem processes respond to climate change should manipulate both temperature and precipitation, among other drivers (CO_2_ fertilization, nitrogen deposition, etc), to investigate their interactive effects and mechanisms underlying the non-linear responses, which has been a focus of several recent studies [36,61,64,69–71]. Particularly, the nonlinear responses of carbon cycles to warming may not only be regulated by year-to-year precipitation amount, but also by changes in timing, intensity, and frequency of precipitation events [47,72,73], which deserves further study if more detailed datasets are available with high spatial and temporal resolutions. Moreover, the precipitation-mediated effects on temperature sensitivity of plant growth and carbon cycles should also be considered in current state of the art land surface models to better represent the nonlinearity of carbon cycle responses and feedbacks to the climate.
